# Mechanical stretching determines the orientation of osteoblast migration and cell division

**DOI:** 10.1007/s12565-023-00716-8

**Published:** 2023-04-06

**Authors:** Fumiko Takemoto, Yoko Uchida-Fukuhara, Hiroshi Kamioka, Hirohiko Okamura, Mika Ikegame

**Affiliations:** 1 Department of Oral Morphology, Graduate School of Medicine, Dentistry and Pharmaceutical Sciences, Okayama University, 2-5-1 Shikata-cho, Kita-ku, Okayama, 700-8525 Japan; 2 Department of Orthodontics, Graduate School of Medicine, Dentistry and Pharmaceutical Sciences, Okayama University, 2-5-1 Shikata-cho, Kita-ku, Okayama, 700-8525 Japan

**Keywords:** Cell alignment, Cell division, Mechanical stress, Migration, Osteoblasts

## Abstract

Osteoblasts alignment and migration are involved in the directional formation of bone matrix and bone remodeling. Many studies have demonstrated that mechanical stretching controls osteoblast morphology and alignment. However, little is known about its effects on osteoblast migration. Here, we investigated changes in the morphology and migration of preosteoblastic MC3T3-E1 cells after the removal of continuous or cyclic stretching. Actin staining and time-lapse recording were performed after stretching removal. The continuous and cyclic groups showed parallel and perpendicular alignment to the stretch direction, respectively. A more elongated cell morphology was observed in the cyclic group than in the continuous group. In both stretch groups, the cells migrated in a direction roughly consistent with the cell alignment. Compared to the other groups, the cells in the cyclic group showed an increased migration velocity and were almost divided in the same direction as the alignment. To summarize, our study showed that mechanical stretching changed cell alignment and morphology in osteoblasts, which affected the direction of migration and cell division, and velocity of migration. These results suggest that mechanical stimulation may modulate the direction of bone tissue formation by inducing the directional migration and cell division of osteoblasts.

**Supplementary Information:**

The online version contains supplementary material available at 10.1007/s12565-023-00716-8.

## Introduction

Mechanical stimulation affects various cellular activities, such as proliferation, differentiation, morphology, and migration, which are important for tissue development and repair (Li et al. 2009; Faust et al. 2011; Sun et al. 2022). Mechanical stimulation is critical for bone formation and remodeling to facilitate adaptation to the surrounding environment (Frost 2003; Tyrovola 2015). Osteoblasts, which are responsible for bone formation, respond to mechanical stimuli (Robling and Turner 2009; Gerosa and Lombardi 2021). Changes in the alignment and migration properties of osteoblasts induced by mechanical stimulation are considered to contribute to directional bone matrix formation and bone remodeling.

The response of osteoblasts to mechanical stress has been studied using cell culture techniques by applying various mechanical stimuli, such as substrate strain, fluid shear stress, and hydrostatic pressure (Sato et al. 2015; Manokawinchoke et al. 2021). Many studies have demonstrated that mechanical stimulation induces changes in osteoblast morphology and alignment. Continuous stretching induced cell alignment parallel to the stretch direction (Li et al. 2020). In contrast, cyclic stretching induced cell alignment perpendicular to the stretch direction (Matsugaki et al. 2013; Matsuzaka et al. 2021). Such a difference in the arrangement direction induced by different stretch modes is also observed in fibroblasts (Weidenhamer and Tranquillo 2013; Huang et al. 2013), and vascular endothelial cells (Sato and Ohashi 2005; Barron et al. 2007; Figueroa et al. 2014). However, limited studies exist regarding the effect of mechanical stimulation on the migratory properties of osteoblasts (Shirakawa et al. 2014; Riehl et al. 2017). The cell alignment and migration of osteoblasts are thought to contribute to bone matrix formation (Hosaki-Takamiya et al. 2016). Therefore, it is important to understand the changes in migration properties, such as direction, distance, velocity of migration, and collective cell migration induced by mechanical stimuli.

Cell migration is driven by the dynamics of the actin cytoskeleton and focal adhesions (Gardel et al. 2010; Kuo 2013), which are major mechanosensors. Stress fibers, primarily composed of actin and myosin II, sense the stiffness or mechanical tension of the extracellular matrix from focal adhesions and are reorganized, resulting in changes in cell alignment and morphology (Boccafoschi et al. 2010; Chatterjee et al. 2022). Differences in the shapes of substrates can control the alignment and morphology of cells adhering to them, as well as their migration direction and velocity (Cheng et al. 2021). However, little is known regarding the effects of mechanical stimulation-induced changes in cell morphology on migration properties.

Here, we investigated how cell alignment and morphology induced by continuous or cyclic stretching affect the migration properties of osteoblasts after stretching removal.

## Materials and methods

### Cell culture

The mouse preosteoblastic cell line MC3T3-E1 (purchased from RIKEN, Saitama, Japan) was cultured in alpha minimum essential medium (Gibco BRL, Rockville, MD, USA) supplemented with 10% heat-inactivated fetal bovine serum and 1% antibiotic solution (Nacalai Tesque, Kyoto, Japan) at 37℃ and 5% CO_2_.

### Application and conditions of stretch

**Fig. 1:**
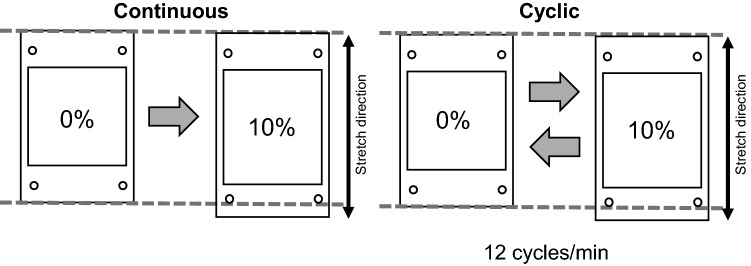
Diagram representing the cell culture chambers. The chambers are stretched in the direction indicated by the double-headed arrows at 10% magnitude, continuously or cyclically (12 cycles/min)

The cells were seeded at a density of 5 × 10^4^ cells/chamber in a 20 × 20 mm silicone hybrid chamber (SC4Ha; Menicon Life Science, Nagoya, Japan) coated with type I collagen (Cellmatrix Type I-C; Nitta Gelatin Inc., Osaka, Japan). After pre-incubation for 24 h under static conditions, prior to stretch application, the medium was changed to a differentiation medium containing 50 μM ascorbic acid (Sigma-Aldrich, St Louis, MO, USA) and 2 mM β-glycerophosphate (Tokyo Kasei, Tokyo, Japan). Uniaxial stretch was applied using the ShellPa^®^ mechanical stretch system (Menicon Life Science). In the continuous stretch group, the cells were stimulated by stretching the silicon chamber at 10% magnitude continuously. In the cyclic stretch group, cells were stimulated by stretching the chamber at 10% magnitude with a frequency of 12 cycles/min (2.5 s stretch and 2.5 s release repeated cyclically) (Fig. 1). Following continuous or cyclic stretch stimulation for 6 h, cells were incubated under appropriate conditions for up to 48 h. In the control group, the medium was changed to the differentiation medium, and the cells were incubated as described above for 6 h without stretching.

### F-actin staining

After stretching, the cells on the flexible membrane were washed twice with phosphate-buffered saline (PBS), fixed with 4% paraformaldehyde solution for 15 min at room temperature, washed again in PBS, and then permeabilized with 0.1% Triton X-100 in PBS for 1 min on ice. Thereafter, F-actin was stained using Actin-Tracker Green solution (Invitrogen, Thermo Fisher Scientific Waltham, MA, USA) according to the manufacturer’s instructions.

### Cell orientation and morphology

Cells at 0 or 24 h after stretch removal were stained for F-actin and observed under a confocal laser scanning microscope. To quantify cell orientation, the angle between the major axis of the cell and the stretch direction was measured using CellProfiler software (Broad Institute, Cambridge, MA, USA). To analyze the cell morphology, the cell aspect ratio (major axis length/minor axis length) was obtained using NIH ImageJ Fiji. The measurements were repeated independently by three people.

### Cell migration direction and velocity

Cells were stained with Hoechst staining (Dojindo, Kumamoto, Japan) for 30 min and cultured under appropriate conditions. Time-lapse recordings of the cells were acquired every 30 min for 24 h using multi-purpose microscope, BZ-X710 (Keyence Engineering Corporation, Osaka, Japan). Time-lapse images were analyzed to determine the direction of cell migration, and the mean migration velocity was analyzed using a BZ-X Analyzer (Keyence Software Corporation). The cell migration direction is presented as the ratio of the migration length on the Y-axis to the migration length on the X-axis. The Y-axis is defined as the stretch direction. The mean migration velocity is presented as the total actual distance traveled by the cell divided by the recording time. Data for each checkpoint were acquired from cell migration distances recorded during the 6 h preceding the checkpoint.

### Cell division direction

The direction of cell division was defined as the angles between the cell division axis and the stretch direction. Time-lapse images of the cells in telophase, when the cytoplasm divides, were evaluated using ImageJ Fiji.

### Statistics

Data are presented as the mean ± standard error of the mean (SEM), unless otherwise specified. One-way ANOVA followed by Bonferroni’s multiple comparison test was used to determine whether the cell alignment angle, aspect ratio, cell migration direction and velocity changed with stretch condition. The numbers of cells for each analysis were indicated in figure legends. All calculations were performed using the statistical software package SPSS 28.0 for Windows (SPSS Japan, Tokyo, Japan). Statistical significance was considered at a *P*-value < 0.05 (Uchida-Fukuhara et al. 2022).

## Results

### Different cell alignment after continuous and cyclic stretchings

**Fig. 2:**
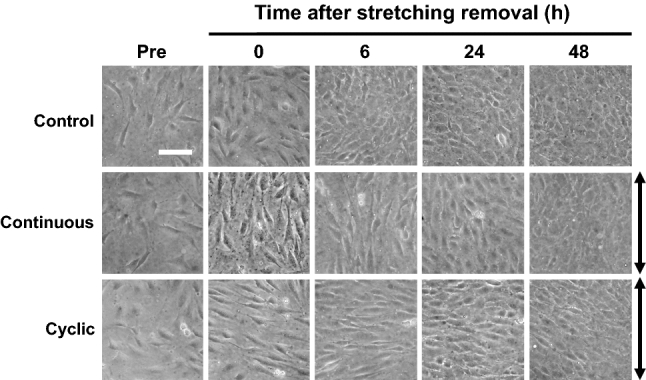
Morphological changes induced by continuous and cyclic stretching. Phase-contrast microscopy images of MC3T3E-1 cells before stretching (Pre), and after stretching removal. All groups showed random cell orientation before stretching. In the continuous group, the cells showed a parallel orientation to the stretch direction at 0 and 6 h after stretching removal. In the cyclic stretch group, the cells showed a perpendicular orientation at 0, 6, and 24 h. In the control groups, the cells showed no specific orientation. Top: control group, middle and bottom: stretch groups, double-headed arrows: the stretch direction, scale bar: 100 μm

Initially, two different types of mechanical stretch were applied to the MC3T3-E1 cells: continuous or cyclic stretching (12 cycles/min) for 6 h (Fig. 1). Cell morphology and alignment were observed pre-stretching, and at 0, 6, 24, and 48 h after stretching removal (Fig. 2). At all observation time points, the cells in the control group showed a random alignment of cells (Fig. 2, top panels). Immediately after stretch removal, cells in the continuous group aligned parallel to the stretch direction (Fig. 2, middle panels), whereas in the cyclic group, cells aligned perpendicular to the stretch direction (Fig. 2, bottom panels). Cell orientations were observed for up to 6 and 24 h, in the continuous and cyclic groups, respectively.

### Quantification of the cell orientation and cell morphology

**Fig. 3:**
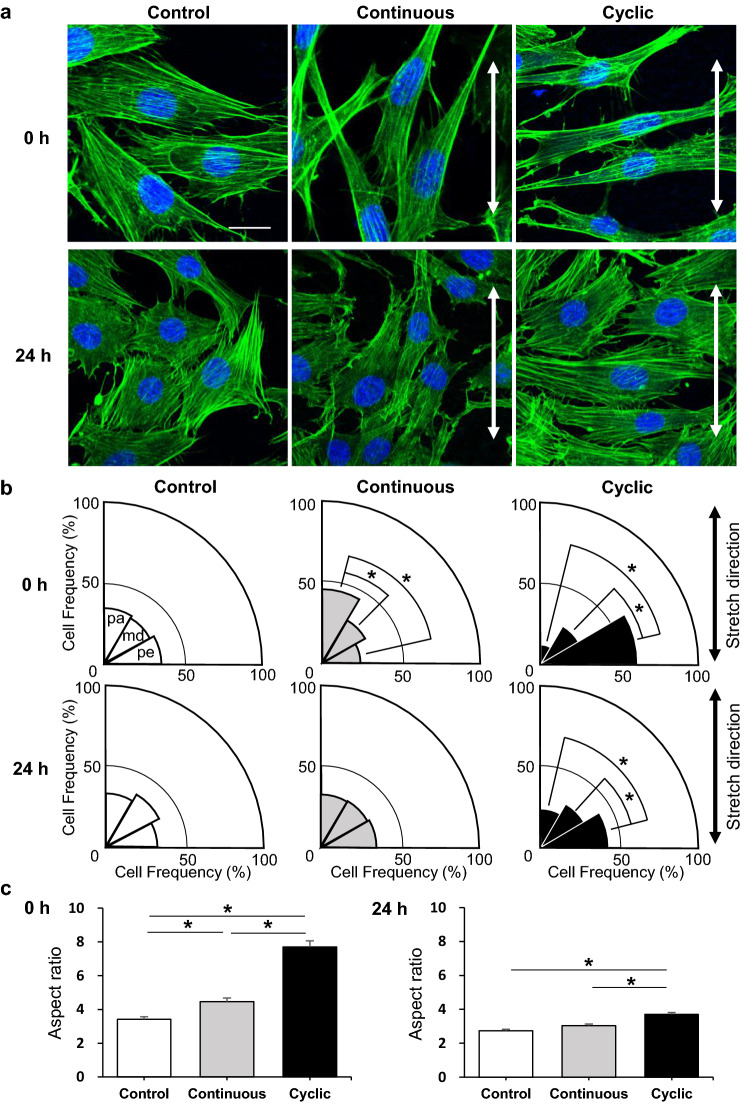
MC3T3E-1 cells alignment and morphology. **a** Confocal laser scanning microscopy images of F-actin-stained cells, immediately (0 h) and at 24 h after stretch removal. Green: F-actin, blue: nuclei stained with DAPI, double-headed arrows: the stretch direction, scale bars: 20 μm. **b** Histograms for distribution of the cell alignment angles which were defined as the angles between the major axis of the cell and the stretch direction (double-headed arrows). The angles were divided into the following three groups for evaluation: pa, parallel (from 0° to 30°); md, middle (above 30°, up to 60°); pe, perpendicular (above 60°, up to 90°). Measurement was performed on three fields of view observed with a × 10 objective lens. The numbers of evaluated cells: control, 0 h = 365, 24 h = 320; continuous, 0 h = 487, 24 h = 524; cyclic, 0 h = 424, 24 h = 647. Data are presented as the ratio (%) to the total cell number. **c** The aspect ratio (major axis/minor axis) of MC3T3-E1 cells in each group, immediately (0 h) and at 24 h after stretching removal. The aspect ratio was higher in the cyclic group than in the other groups, which means that the cell morphology in the cyclic group was more elongated. The numbers of evaluated cells: control, 0 h = 226, 24 h = 243; continuous, 0 h = 201, 24 h = 306; cyclic, 0 h = 214, 24 h = 333. Data are presented as the mean ± SEM. *P < 0.05, One-way ANOVA followed by Bonferroni’s multiple comparison test

F-actin staining images show that most of the stress fibers aligned in the direction parallel to the major axis of the cells in each group, which was especially apparent in the stretch groups right after stretch removal (0 h) (Fig. 3a). The outline of the cells stained for F-actin was recognized by CellProfiler and the cell orientation was quantified as the angle between the major axis of the cell and the stretch direction. The angles from 0 to 90 degrees were divided into 10-degree increments and the number of cells belonging to each angle section was calculated (Supplementary Fig. 1). We further divided the cell alignment angles into three groups: parallel, middle, perpendicular; and analyzed whether there are any significant differences among them (Fig. 3b). The cells in the control group did not indicate a specific direction at any observation time point (average angle, 46°). Immediately after stretching removal (0 h), in the continuous group, the number of cells in the parallel group was significantly higher than those in other groups, indicating that cells were aligned nearly parallel to the stretch direction (average angle, 37°). In contrast, in the cyclic group, the cells were aligned nearly perpendicular to the stretch direction (average angle, 61°). At 24 h after stretch removal, in the continuous group, the cells were aligned randomly (average angle, 45°), but in the cyclic group, many of the cells still maintained in a perpendicular orientation to the stretch direction (average angle, 52°).

The cells in the control group exhibited a polygonal shape, whereas the cells in both stretch groups appeared elongated in each alignment direction. Therefore, we attempted to quantify cell morphology from F-actin-stained images. The aspect ratio of each cell was obtained by dividing the length of the major axis by the minor axis (Fig. 3c). Immediately after stretching removal, the cell aspect ratio in the cyclic group was significantly higher than that in the other groups and was relatively maintained after 24 h (Fig. 3c).

### Analysis of cell migration and cell division

**Fig. 4:**
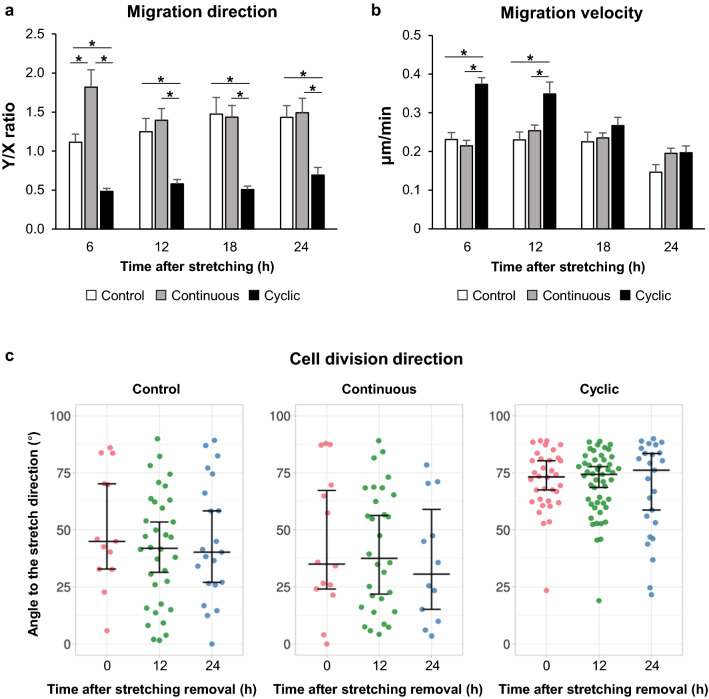
MC3T3E-1 cells migration properties and cell division direction. **a** The cell migration direction was presented as the ratio of the Y-axis migration length to the X-axis migration length. The Y-axis is defined as the stretch direction. **b** The mean migration velocity within each 6 h checkpoint. Data are presented as the mean ± SEM. *P < 0.05, One-way ANOVA followed by Bonferroni’s multiple comparison test. The numbers of evaluated cells for **a** and **b**: control, n = 16; continuous, n = 23; cyclic, n = 21. Three random fields per sample were examined under a × 20 objective. Each experiment was repeated at least three times. **c** The cell division direction was defined as the angle between the cell division axis and the stretch direction. Graphs show the data as jittered dots. The numbers of evaluated cells: control, 0 h = 13, 12 h = 34, 24 h = 21; continuous, 0 h = 14, 12 h = 30, 24 h = 12; cyclic, 0 h = 34, 12 h = 49, 24 h = 25. The horizontal bars in the middle indicate the median values for each group: control, 0 h = 45.0, 12 h = 41.9, 24 h = 40.2; continuous, 0 h = 35.0, 12 h = 37.5, 24 h = 30.6; cyclic, 0 h = 73.2, 12 h = 74.4, 24 h = 76.2. Vertical bars indicate 95% confidence intervals for each median

After stretch removal, cell migration was tracked using time-lapse recording. The migration direction was indicated by the ratio of the migration length in the Y-axis to the migration length in the X-axis. Thus, a Y/X ratio greater than one means more movement along the Y-axis, and less than one means more movement along the X-axis. The direction of cell migration was consistent with that of cell alignment. In the control group (Fig. 4a, white bars), the cells migrated equally in both the X- and Y-axes at 6 h, but slightly more in the Y-axis direction at 12 h and later after stretch removal. In the continuous group (Fig. 4a, gray bars), the cells were more likely to move in the Y-axis direction (Y/X ratio = 1.8), parallel to the stretch direction, at 6 h after stretching. However, at 12 h and later, they migrated in a direction similar to that of the control group. In the cyclic group (Fig. 4a black bar), the cells migrated approximately twice as much in the X-axis direction up to 24 h after stretch removal (Y/X ratio = 0.5–0.7). In Fig. 4b, the values of migration velocity in the continuous group were similar to those in the control group at all time points. However, in the cyclic group, the values of migration velocity were significantly greater at 6 and 12 h compared to those in the other groups (Fig. 4b).

To analyze the direction of cell division, we measured the angle between the cell division axis and the stretch direction. In Fig. 4c, the direction of cell division in the cyclic group showed different pattern compared with those in the control and continuous groups. In the control group, the median values ranged from 45.0° to 40.2°, suggesting random cell division direction. In the continuous group, the median values ranged from 35.0° to 30.5°, suggesting the cell division direction was slightly closer to the parallel direction compared to the control group. In contrast, in the cyclic group, most of the cells clearly showed the division direction close to the perpendicular direction to the stretching, with median values ranging from 73.2° to 76.2°. The distribution range in the cyclic group was narrower than those in the other groups, and gradually widened over time (Fig. 4c).

## Discussion

In this study, we investigated the cellular morphology and migration after removing the continuous and cyclic stretching. We clarified that mechanical stretching can control not only the cell morphology but also the migration properties. The MC3T3-E1 cells were aligned parallel to the stretch direction in the continuous group and perpendicular to the stretch direction in the cyclic group, and maintained their alignment up to 6 and 24 h, after stretching removal, respectively. We also found that mechanical stretching regulated the direction of cell migration and division, and further increased migration velocity. These correlated with the changes in the cell alignment direction and the degree of morphological deformation.

The importance of cell morphology in determining the properties of cell migration has been argued in studies investigating the effect of topography, the shape of a cell scaffold, on cell morphology and migration (Refaaq et al. 2020; Cheng et al. 2021). However, little is known about the effects of cell morphological changes caused by mechanical stimulation on migration properties. The changes in osteoblast alignment and increased migration velocity observed in the present study are largely consistent with results previously obtained in other kinds of cells. Fibroblasts and endothelial cells changed their arrangement direction in the same direction as in this study according to the application of stretching stimulation, and their migration velocity was increased (Eastwood et al. 1998; Kanayama et al. 2008; Zheng et al. 2008; Huang et al. 2013). However, up until now only the direction of cell migration on flow stimulation have been reported (Masuda and Fujiwara 1993; Li et al. 2001; Riehl et al. 2017). Previous researchers demonstrated that fluid flow alters the migration direction and velocity of osteoblasts (Shirakawa et al. 2014; Riehl et al. 2017). However, the effects were temporary, and after a short period, the cells became unresponsive to the stimuli (Riehl et al. 2017). Here, we showed that the morphological changes in cell alignment and aspect ratio induced by different modes of stretching were correlated with the characteristics of cell migration, and the effects persisted for 6 or 24 h after stretching removal. The cells in the continuous and cyclic groups aligned in different directions and migrated in aligned directions. Moreover, cell migration velocity and aspect ratio were greater in the cyclic group compared to those in the other groups. When cells migrate faster, they possess a relatively elongated shape (Refaaq et al. 2020; Cheng et al. 2021). This suggests that morphological changes in osteoblasts induced by mechanical stretching are correlated with the direction and velocity of migration. Our report is the first to examine the morphological changes and migratory properties of cells in detail after applying different modes of stretch stimulation.

The difference of cell orientation between the continuous and cyclic stretch modes has been discussed in several papers. It is widely believed that cells, when given a stretch stimulus, rearrange and align their cytoskeleton in the minimum strain direction (Matsugaki et al. 2013). Our results were also considered to follow that rule. We also found that the maintenance time of cell alignment and migration direction and velocity differed between stimulation modes. The cells in the cyclic group stayed in the same alignment and migrate in that direction for a longer time in higher velocity than the cells in the continuous group. At 24 h after stretch removal, the cells in the continuous group showed random alignment. In contrast, the cells in the cyclic group maintained relatively similar cell alignment as immediately after stretching. This phenomenon may be due to the difference in the stimulus intensity. It has been reported that cyclic stretch stimulates cellular responses in osteoblasts more strongly than continuous stretch (Winter et al. 2003). Moreover, cyclic stress on integrins in osteoblasts elicited higher maximal calcium responses and activated focal adhesion kinase more effectively compared with continuous stress (Pommerenke et al. 2002). Mechanical stimulation on integrins also activates several factors involved in migration, such as Rho family GTPases (Raftopoulou and Hall 2004). Activation of these factors by stretching stimuli is known to promote cell forward elongation and posterior contraction, resulting in cell migration (Gardel et al. 2010). Thus, the repeated stimulation by cyclic stretch is expected to increase the amount of these biological signals, resulting in the sustained cell alignment and migration direction with higher migrating velocity.

The direction of cell division is important for the tissue development and growth patterns (Gillies and Cabernard 2011; Castanon and González-Gaitán 2011). Therefore, we further examined the effect of stretching on the direction of cell division in osteoblasts. In the cyclic group, the direction of cell division almost coincided with the direction of cell alignment, which is the direction of major axis, in other word the long axis of the cells. This result is well consistent with the “long axis rule” (Finegan and Bergstralh 2019), which states that cells divide along their longitudinal axis. Nevertheless, in the continuous group, cells had a weak tendency to divide in the alignment direction. This can be explained by the fact that the mitotic spindle, which determines the direction of cell division, is highly sensitive to the cell aspect ratio. Fernandez reported that the direction of cell division depends on the degree of cell deformation induced by mechanical share stress: the drastic cell elongation caused by high-frequency shear strain strongly biased the mitotic spindle orientation (Fernandez et al. 2011). In our study, cell deformation in the continuous group was smaller than that in the cyclic group. Therefore, not only the changes in cell alignment, but also the changes in cell morphology due to mechanical stimulation are considered to be important factors in regulating the direction of cell division.

In conclusion, we revealed that osteoblasts migrate in the direction of their alignment induced by mechanical stretching, further accelerating the migration velocity. In addition to that, cell division proceeds in the direction of cell alignment. These results suggest that mechanical stimulation modulates the direction of osteoblast migration and division, thereby possibly influencing the direction of bone tissue formation.

## Supplementary Information

Below is the link to the electronic supplementary material.

Supplementary file1 (PPTX 72 KB)

## Data Availability

The datasets analyzed during the current study are available from the corresponding author on request.
