# Supplementary material for: Mechanical stretching determines the orientation of osteoblast migration and cell division
Source: Anat Sci Int. 2023 Apr 6;98(4):521–8. doi: 10.1007/s12565-023-00716-8 (PMC10366257; doi:10.1007/s12565-023-00716-8)
Supplement: Supplementary file 1 — Supplementary file1 (PPTX 72 KB) [file 12565_2023_716_MOESM1_ESM.pptx]

## Slide 1
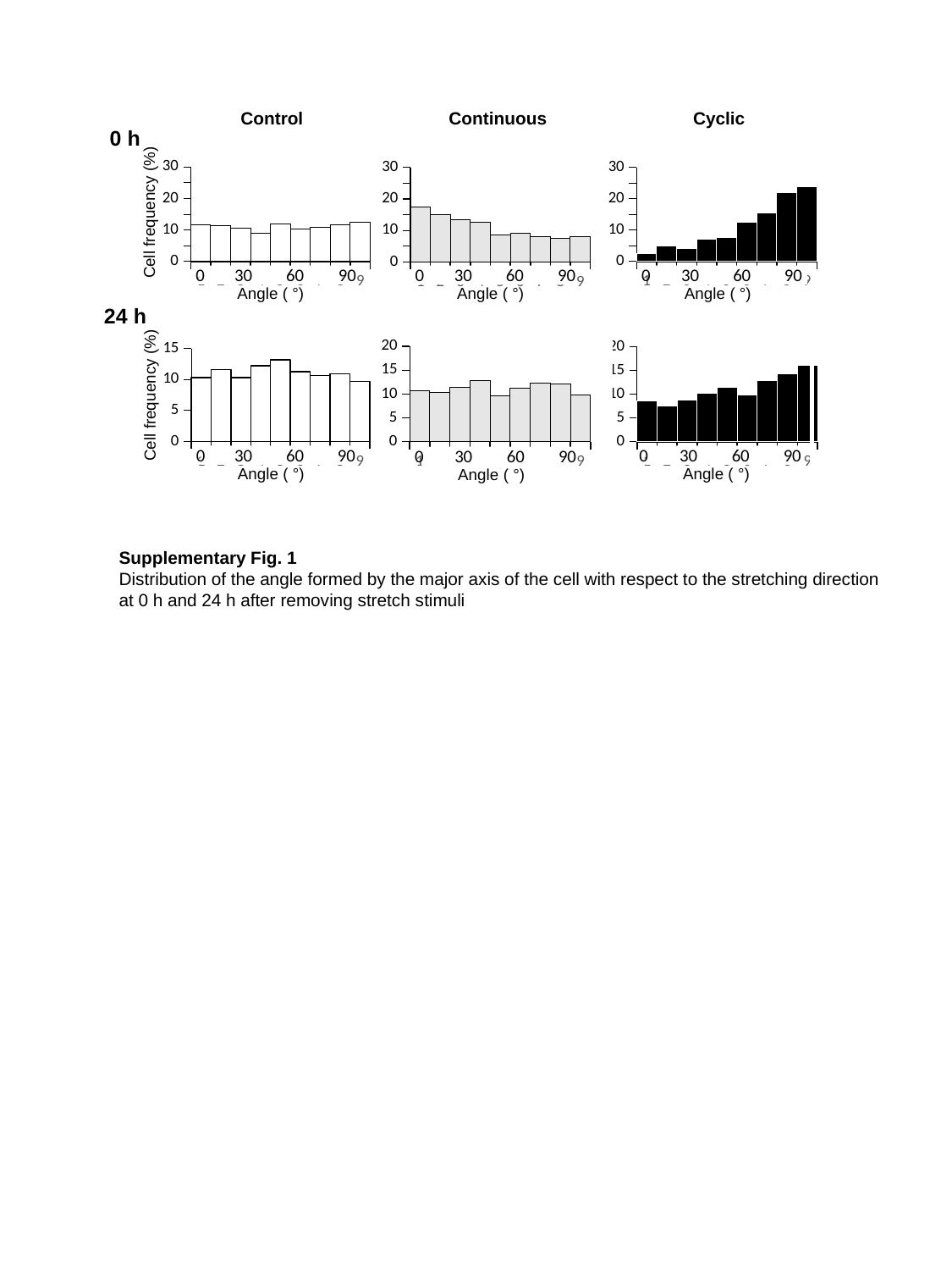

Control
Continuous
Cyclic
0 h
### Chart
| Category | |
|---|---|
### Chart
| Category | |
|---|---|
### Chart
| Category | |
|---|---|Cell frequency (%)
0
30
60
90
Angle ( °)
0
30
60
90
Angle ( °)
0
30
60
90
Angle ( °)
24 h
### Chart
| Category | |
|---|---|
### Chart
| Category | |
|---|---|
### Chart
| Category | |
|---|---|Cell frequency (%)
0
30
60
90
Angle ( °)
0
30
60
90
Angle ( °)
0
30
60
90
Angle ( °)
Supplementary Fig. 1
Distribution of the angle formed by the major axis of the cell with respect to the stretching direction at 0 h and 24 h after removing stretch stimuli
